# Developing machine learning systems worthy of trust for infection science: a requirement for future implementation into clinical practice

**DOI:** 10.3389/fdgth.2023.1260602

**Published:** 2023-09-27

**Authors:** Benjamin R. McFadden, Mark Reynolds, Timothy J. J. Inglis

**Affiliations:** ^1^ School of Physics, Mathematics and Computing, University of Western Australia, Perth, WA, Australia; ^2^ Western Australian Country Health Service, Perth, WA, Australia; ^3^ School of Medicine, University of Western Australia, Perth, WA, Australia; ^4^ Department of Microbiology, Pathwest Laboratory Medicine, Perth, WA, Australia

**Keywords:** machine learning, trustworthy machine learning, infection science, machine learning implementation, clinical practice, infectious disease, infection, machine learning systems frontiers

## Abstract

Infection science is a discipline of healthcare which includes clinical microbiology, public health microbiology, mechanisms of microbial disease, and antimicrobial countermeasures. The importance of infection science has become more apparent in recent years during the SARS-CoV-2 (COVID-19) pandemic and subsequent highlighting of critical operational domains within infection science including the hospital, clinical laboratory, and public health environments to prevent, manage, and treat infectious diseases. However, as the global community transitions beyond the pandemic, the importance of infection science remains, with emerging infectious diseases, bloodstream infections, sepsis, and antimicrobial resistance becoming increasingly significant contributions to the burden of global disease. Machine learning (ML) is frequently applied in healthcare and medical domains, with growing interest in the application of ML techniques to problems in infection science. This has the potential to address several key aspects including improving patient outcomes, optimising workflows in the clinical laboratory, and supporting the management of public health. However, despite promising results, the implementation of ML into clinical practice and workflows is limited. Enabling the migration of ML models from the research to real world environment requires the development of trustworthy ML systems that support the requirements of users, stakeholders, and regulatory agencies. This paper will provide readers with a brief introduction to infection science, outline the principles of trustworthy ML systems, provide examples of the application of these principles in infection science, and propose future directions for moving towards the development of trustworthy ML systems in infection science.

## 1. Introduction

Research relating to the application of machine learning (ML) has continued to grow in a range of subject areas. Particularly, the use of ML continues to increase in high-stake environments where data is being used to support humans in decision-making scenarios and in some cases, completely removing human involvement. In healthcare, data driven processes are becoming more prominent along with the application of ML. This is evident by the growing application of ML in many areas of healthcare including radiology, cardiology, neurology, and general hospital use (1). This is the result of the increased availability and development of ML techniques, and the infrastructure that supports the collection, storage, and access to large volumes of data in the healthcare setting. Different healthcare environments have their own set of unique challenges, with the effective care of patients relying on a number of human teams from different departments, especially in the hospital setting, which relies on strong interdisciplinary teamwork for processes to operate efficiently and effectively. Infection science (IS) is a discipline that includes clinical microbiology,public health microbiology, mechanisms of microbial disease, and antimicrobial countermeasures (2). In particular, the identification and subsequent treatment of infectious disease as a result of infection due to bacteria, viruses, fungi, and parasites, in addition to veterinary and environment microbiology. The clinical laboratory setting plays a significant role in IS, with focus directed towards supporting the treatment of patients with a range of laboratory processes and tests from various departments including clinical chemistry, hematology, clinical microbiology, and molecular pathology. All of these have an important role in the decision making process for medical teams when determining the best course of treatment for patients in the clinical setting. The clinical laboratory also provides the data used by public health authorities to support disease control and management in the community including epidemics, pandemics, and ongoing public health concerns (3). The importance of IS has existed throughout history, combating various pandemics, most recently with the SARS-CoV-2 (COVID-19) pandemic (4), and ongoing into the future, with emerging infectious disease, the increasing rise of antimicrobial resistance (AMR) (5), and the global burden of sepsis and bloodstream infections (BSIs) (6). Recently, there has been a significant increase in the number of research projects dedicated to the application of ML techniques to address problems in IS, focusing on patient care in the hospital setting, clinical laboratory workflows and processes, public health management, and the interplay between these areas. ML techniques utilise data in a variety of forms from different sources including electronic health records (EHRs), laboratory information systems (LIS), clinical notes, and images. However, despite the increasing interest of ML in IS, and the growing number of applications in this area, a significant gap remains between the number of ML models in research and real-world clinical environments. This disparity is largely attributed to the absence of trustworthy ML systems. Research relating to the development of these systems for the IS domain that meet the requirements of users, regulatory agencies, and the stakeholders is significantly underrepresented in the literature.

## 2. Machine learning and infection science

ML is a type of artificial intelligence (AI), described as a set of methods/algorithms that learn patterns from data, utilising these patterns to produce predictions (7). ML techniques have increasingly been applied in medicine and healthcare. Recently, research relating to the application of ML to IS has become more prominent, with ML technology having the potential to improve several aspects of IS including treatment, diagnosis, and management of patients, optimising and improving clinical laboratory workflows, and improving public health surveillance. Implications of applications in these area include improvement of patient outcomes, optimising for timely patient treatment, reduction in costs and unnecessary resource expenditure, and early identification and mitigation of emerging infectious disease to support readiness for potential public health emergencies. The increase in ML applications for IS was made apparent during the height of the COVID-19 pandemic, where a significant body of research was dedicated to the application of ML for problems associated with COVID-19. Contemporary reviews of the literature have demonstrated the increasing application of ML for IS, with ML being applied across a range of areas. These reviews ranging from 2020–2022 have been referenced in Table 1. IS involves the hospital, clinical laboratory, and public health authorities. All of these areas have an important role in the management and treatment of disease associated with pathogenic organisms. In the hospital setting, infectious disease is of considerable concern to patient health outcomes. In particular, sepsis, currently defined as a life threatening organ dysfunction due to a dysregulated immune response to infection as a result of pathogenic organisms (22), is a significant area of interest. Sepsis, BSIs, and the continuing rise of AMR, a process that occurs when pathogens change over time and do not respond to typical treatment, are all threats to global population, with an estimated 4.95 million (95% UI 3.62–6.57) deaths as a result of drug-resistant infections, with 1.27 million of those deaths resulting directly from drug resistance in 2019 (23). Patients with BSIs present in the emergency department (ED), intensive care unit (ICU), and postoperative care environments as a result of surgical site infections. Other hospital acquired infections, particularly central line-associated bloodstream infections (CLABSI) are also a risk to patients. The hospital setting relies on physicians to identify patients with suspected infections, and recommend the appropriate course of action with cooperation from the clinical laboratory, which is responsible for performing the tests utilised for patient care. The different areas of the clinical laboratory have their own responsibilities and respective objectives. These areas include hematology, clinical chemistry, and clinical microbiology. Data from the clinical laboratory is often used to identify infection trends and patterns of AMR over time, an area often referred to as microbial surveillance. The role of public health is to make recommendations based on these trends and respond to emerging threats. This was made apparent most recently with the role of public health authorities in implementing measures to reduce the incidence of COVID-19 during the pandemic (24). In the future, public health will continue to focus on emerging infectious diseases, BSIs, and patterns of AMR. Challenges for implementing ML systems into clinical practice have been previously identified including algorithmic bias, clinical applicability, and dataset shift (25). Despite the promise of ML applications in IS, there is an incomplete understanding regarding the barriers to ML implementation in practice. However, trust in ML systems from the perspective of users, stakeholders, and regulatory agencies has a significant role in the adoption of ML systems in IS. The remainder of this paper will explore this perspective.

**Table 1:** Recent literature reviews relating to the application of machine learning in infection science.

Review title	Year	Focus area
Artificial intelligence and machine learning assisted drug delivery for effective treatment of infectious diseases (8)	2021	Drug delivery
Evolving Applications of Artificial Intelligence and Machine Learning in Infectious Diseases Testing (9)	2021	Infectious disease testing
Machine learning for clinical decision support in infectious diseases: a narrative review of current applications (10)	2020	Decision support systems
Machine learning in infection management using routine electronic health records: tools, techniques, and reporting of future technologies (11)	2020	General infection management
Machine learning applications for COVID-19: a state-of-the-art review (12)	2021	COVID-19
Significant applications of machine learning for COVID-19 pandemic (13)	2020	COVID-19
Applications of machine learning and artificial intelligence for Covid-19 (SARS-CoV-2) pandemic: a review (14)	2020	COVID-19
Role of machine learning techniques to tackle the COVID-19 crisis: systematic review (15)	2021	COVID-19
Early prediction of sepsis in the ICU using machine learning: a systematic review (16)	2021	Sepsis in the ICU
Viral outbreaks detection and surveillance using wastewater-based epidemiology, viral air sampling, and machine learning techniques: a comprehensive review and outlook (17)	2022	Surveillance and epidemiology
Recent evolutions of machine learning applications in clinical laboratory medicine (18)	2021	Clinical laboratory
Machine learning in the clinical microbiology laboratory: has the time come for routine practice? (19)	2020	Clinical laboratory
Applications of machine learning in routine laboratory medicine: current state and future directions (20)	2022	Clinical laboratory
Use of artificial intelligence in infectious disease (21)	2020	Infectious disease: diagnosis, transmission, response to treatment, and resistance

## 3. Principles of trustworthy machine learning systems

Recently, the principles associated with trustworthy AI have become more of a focus in the research community as ML becomes increasingly applied in high-stakes environments such as finance, defence, and healthcare. This includes identifying the requirements for the development of trustworthy ML systems, and establish pathways to adoption for ML systems in practice.

The foundational definition of trustworthy AI was presented by the independent high-level expert group on artificial intelligence (HLEG), established by the European Commission (26). They created an ethics guideline for trustworthy AI which develops a human-centric approach to AI and includes seven key requirements that should be considered when developing ML systems to ensure they are considered trustworthy. These include the following:

• Human agency and oversight
• Technical robustness and safety
• Privacy and data governance
• Transparency
• Diversity, non-discrimination and fairness
• Societal and environmental well-being
• Accountability

The implementation, relevance, and specific interpretations of the trustworthy AI requirements ultimately depends on the specific domain and context in which the ML system is being developed and deployed (27). The guidelines presented by the HLEG outline the components of trustworthy AI, but they do not provide specific direction regarding the implementation of these guidelines in practice. Furthermore, there are multiple definitions of trust in the context of ML systems, with a significant amount of the literature dedicated to the theories, philosophies, and psychological aspects of trust. However, these definitions do not necessarily assist in the design, engineering, and development of trustworthy ML systems (28), and do not provide guidance surrounding specific details of implementation. This is further complicated by the presence of sub-domains within different application areas, which is the case in healthcare. Therefore, when referring to the development of trustworthy ML systems, more practical definitions are essential to provide a foundation for the practical development of ML systems worthy of trust, such as the one provided by Varshney, *A trustworthy machine learning system is one that has sufficient basic performance, reliability, human interaction, and aligned purpose* (29).

In healthcare, it is the responsibility of the stakeholders in each of the unique domains to develop their own specific set of best practices that are appropriate for their own context. When developing ML systems worthy of trust, it is important to consider the entire lifecycle of the ML system. This involves data collection and processing, ML model development, and subsequent deployment and monitoring of the model. ML model development involves training, testing, and validation of the model, and deployment requires taking the model from the research, to the real world environment. This is often achieved by providing an interface to enable users to interact with the ML system, through a client-side application such as a web or mobile application, or through the use of an application programming interface (API) deployed through the use of cloud computing resources or on premise infrastructure. The principles of trustworthy ML systems need to be integrated at each of the stages in this ML system development and deployment lifecycle. This has been previously identified by Li et al. (30), where the authors provided a survey of the practices used for building trustworthy AI systems, organising them based on each of the elements of the typical ML lifecycle including data preparation, the design and development of ML models, the deployment into production environments, and the workflow for continuous maintenance of the system. Some of these practices include the integration of bias mitigation strategies, privacy preservation techniques, approaches to ensure adversarial robustness, interpretable ML techniques and algorithms, and federated learning. They also highlighted the importance of TrustAIOps, a workflow which builds upon both the principles of DevOps (development operations) and MLOps (machine learning operations).

The recent attempts at establishing the principles, and guidelines for trustworthy AI systems has also prompted the requirement for methods to assess the overall trustworthiness of a system. An assessment of the trustworthiness of a ML system is necessary to deploy these systems into real world environments. Z-Inspection, introduced by Zicari et al. (31), is one such approach to assessing trustworthy AI, which follows the guidelines established by the HLEG. It consists of three phases, the set up phase, the assess phase, and the resolve phase.The use of this comprehensive protocol has been demonstrated in a number of case studies including predicting the risk of a cardiovascular heart disease (31), machine learning as a supportive tool to recognize cardiac arrest in emergency calls (27), deep learning for skin lesion classification (32), and deep learning system to aid radiologists in estimating and communicating the degree of damage in a patient’s lung as a result of COVID-19 (33). Other recent approaches for assessing whether an AI system is trustworthy includes the trustworthy artificial intelligence implementation (TAII) framework (34), the assessment list for trustworthy AI applications produced by the HLEG (35), and a checklist proposed by Scott et al. which includes 10 questions for clinicians to ask when assessing the viability of ML approaches for use in practice (36).

## 4. Application of trustworthy machine learning principles in infection science

As previously identified, there have been a number of reviews highlighting the application of ML to problems in IS. However, far fewer ML applications implement the principles for developing ML systems that are worthy of trust. The google scholar database was searched to identify research articles that explore and implement the principles and practices of trustworthy machine learning systems for IS. Exact searches for “trustworthy machine learning for infection” did not produce any articles. Articles containing “trustworthy machine learning infection” and “machine learning infection” were searched for. Articles were then manually examined. Examples of studies which have implemented some of these principles based on this search have been included in this section and shown in Table 2. Much of the effort surrounding the development of ML solutions within IS has been directed towards the development of high performing ML models. More recently, effort has been made to include aspects of transparency during the ML modelling stage, by using methods that support interpretability and explainability. These applications align with the transparency requirement for trustworthy AI established by the HLEG. However, research surrounding the development of ML systems for IS which implement practices that support the other requirements is limited. This represents a significant gap in the literature. The increased emphasis on transparency can be identified in recent works regarding the development of ML solutions for problems within the IS domain. For example, interpretability methods are increasingly being applied for problems relating to sepsis. XGBoost was applied for early prediction of in-hospital mortality in critically ill patients with sepsis, along with shapley additive explanations (SHAP) for interpretability (37,38). A modified regularized Weilbull-Cox analysis model was used for prediction of sepsis in ICU patients. This particular model results in an approach which is more interpretable (39). The SHAP method along with partial dependence plots (PDP) were utilised with the gradient-boosted tree algorithm (LightGBM) for risk factor analysis of in-hospital mortality in sepsis survivors with ICU readmission (40). Beyond sepsis, Casiraghi et al. (41) developed an explainable ML decision system which was based on the additive tree approach for COVID-19 risk assessment in emergency departments. Furthermore, Tabaie et al. (42) developed an ML model for the prediction of serious infection in children with central venous lines. The SHAP method was integrated to determine feature importance. Research interest is also increasing in the area of ML deployment and monitoring, an important component of developing trustworthy ML systems. Das et al. (43) developed an ML model for the prediction of COVID-19 community mortality risk in South Korea. They subsequently deployed this model as an online prediction tool. For monitoring of ML systems, data drift is of particular interest. Duckworth et al. (44) utilised explainable ML for detecting emergent health risks for patients in the emergency department during the COVID-19 pandemic. Explainable ML was also used to detect data drift in this case.

**Table 2:** Examples of research papers that implement one or more of the principles of trustworthy ML systems in infection science.

Research title	Year	Overview
Interpretable Machine Learning for Early Prediction of Prognosis in Sepsis: A Discovery and Validation Study (37)	2022	Implemented an XGBoost model for early prediction of in-hospital mortality in critically ill patients with sepsis. Implemented the SHAP method for interpretability of the model
An interpretable machine learning model for accurate prediction of sepsis in the ICU (39)	2018	Used a modified regularized Weilbull-Cox analysis model for prediction of sepsis in ICU patients. This particular model results in an approach which is more interpretable.
Explainable machine learning for early assessment of COVID-19 risk prediction in emergency departments (41)	2020	The authors developed an explainable machine learning decision system which was based on the additive tree approach for COVID-19 risk assessment
An explainable machine learning algorithm for risk factor analysis of in-hospital mortality in sepsis survivors with ICU readmission (40)	2021	Utilised the SHAP method and partial dependence plots (PDP) with a gradient-boosted tree algorithm (LightGBM) for risk factor analysis of in-hospital mortality in sepsis survivors with ICU readmission
Predicting presumed serious infection among hospitalized children on central venous lines with machine learning (42)	2021	The authors introduced a machine learning framework for the prediction of serious infection in children with central venous lines. Explainability was integrated through the use of SHAP values
Predicting CoVID-19 community mortality risk using machine learning and development of an online prognostic tool (43)	2020	The authors trained a logistic regression model to predict mortality among confirmed COVID-19 patients in South Korea. They deployed the model as an online prediction tool named CoCoMoRP
Using explainable machine learning to characterise data drift and detect emergent health risks for emergency department admissions during COVID-19 (44)	2021	The authors utilised explainable ML to characterise data drift and identify emerging health risks for patients in ED during the COVID-19 pandemic

## 5. Discussion

ML based approaches have been producing promising results in IS, providing insight into the utility of ML applications for addressing problems relating to patient management, clinical laboratory workflows, and surveillance and monitoring of public health. The increasing interest in the application of ML in the IS context presents additional opportunities to address the regulatory and system stakeholder concerns regarding the trustworthiness of ML based approaches in practice. The importance of implementing trustworthy ML principles along the entire ML lifecycle, from collection of data through to the deployment, monitoring, and continuous improvement of ML systems is critical to ensuring the success and adoption of these systems in practice. Whilst there has been a significant increase in the number of ML applications in IS, this is not reflected in the real world integration of the models into practice. A significant amount of the work regarding the development of ML systems in healthcare has been directed towards a small number of domains, which is evident by the number of approved AI based systems/devices in the United States of America (US) and Europe (EU). Between 2015 and 2020, 222 and 240 AI/ML devices have been approved in the US and EU respectively (1). The majority of these applications are for radiology, cardiology, neurology, and general hospital use. Only a small number of AI systems have been approved for use in infection science, with a focus on applications in the clinical laboratory. In the US, of the 222 approved devices, 4 are for microbiology (1.8%) and 5 are for hematology (2.25%). In EU, of the 240 systems/devices approved, 9 have been approved for pathology (3.75%), 4 for clinical chemistry (1.67%), 2 for microbiology (0.83%), and 1 for hematology (0.42%). Frameworks for developing trustworthy ML systems have been developed in other areas of healthcare. One such framework has been proposed for opthalmology, which identified five aspects that need to be considered for the training and validation of trustworthy ML systems (45). These five aspects include the quality and completeness of the data used to train the system, the impact of algorithmic bias and capacity to generalize across population groups, explainability of the system to develop trust in the system and enhance clinical utility, system robustness against adversarial attacks and examples, and maintaining, monitoring and updating the system over time in response to user engagement, variations in clinical practice, and data shift to maintain a certain level of performance. This framework incorporates many of the key principles that form the foundation of developing ML systems worthy of trust. More recent works in the IS domain have demonstrated an increased focus on interpretability and explainability of ML models to enhance transparency. Additionally, there is an increasing body of work that is concerned with the deployment of ML models for the purpose of validating performance and establishing pathways to production. Significant opportunities still remain to develop ML systems for IS that adopt principles of trustworthiness in practice, and explicitly discuss their implementation. Developing case studies on integrating ML models into existing workflows for IS, demonstrating the entire end-to-end lifecycle, and capturing both the functional and non-functional requirements for developing trustworthy ML systems is critical. This is particularly important in the clinical laboratory, where processes are highly standardised and quality controlled. More specifically, going beyond model training and testing, to demonstrate deployment, safe monitoring, updating, and the mechanisms for continual learning in ML workflows in the IS context is also of importance. This would also complement an exploration of TrustAIOps, introduced by Li et al. (30). The development of technically robust and safe ML systems is another area that has not been represented in the IS domain. Particularly, ensuring that they are resilient against adversarial examples. Understanding the role that the users and the various stakeholders have in the design and deployment of ML systems is an important area of future work to support the requirement for human agency and oversight. In the context of IS, this includes software engineers, clinical researchers, hospitals, clinical laboratories, the patients, regulatory agencies, and other domains of oversight. Furthermore, it is critical to consider additional user and human factor elements for developing trust in ML systems within the infection science context. For example, previous research has explored methods for user interface design to investigate user trust issues and the creation of trustworthy clinical decision support platforms (46). Additionally, previous work has aimed to identify conceptual models to describe how the definitions and principles associated with trustworthy AI can be effectively communicated in AI systems to enable users to make trust judgements (47). However, linking definitions of trust, principles for the development of trustworthy AI systems, and user perspectives on trust is an ongoing area of research. This is because user trust is context specific, and needs to be addressed for the specific domains in which user-AI systems exist (48). Therefore, addressing trust from the perspective of users is open to future research within the IS domain, particularly the clinical laboratory, where significant potential for human-AI teams exists. This may involve evaluating previous methodologies relating to human factors within this environment, or performing qualitative and/or quantitative assessments on humans within the clinical laboratory environment to determine what components of ML systems are required to foster trust between ML systems and users within this context. Strategies of deployment should also be uniquely considered in the IS context, including important software architecture considerations, and the role that technologies such as cloud, blockchain, and edge computing have in the development of trustworthy ML systems for IS by addressing the requirements for privacy, fairness, and accountability. One particular area that is specific to infection science, is the potentially significant impact that ML based methods have on the clinical laboratory environment at an operations level. This includes how clinical laboratory staff interface with ML systems, the role that human in the loop systems play in this environment, the way regulatory agencies manage this in the clinical laboratory environment, and how the outcomes of the ML systems are effectively explained to laboratory staff and then subsequently communicated to the clinicians that ordered the laboratory tests. Like the other domains of healthcare, IS is unique and therefore requires domain specific approaches to managing the increasing prevalence of ML systems in the critical areas of IS, and ensuring that these systems are worthy of trust in the hospital, clinical laboratory, and public health environments. Furthermore, with the increasing rise of ML applications within healthcare domains, an increase in uncertainty regarding policies and regulations associated with this technology has also become prevalent. Lastly, an increased research focus on the development of trustworthy ML systems should support the development of policies associated with integrating ML systems into healthcare practice. Within infection science particularly, this would result in policies being developed for ML systems that are deployed in the clinical laboratory environment. Such policies may be implemented at a national or local level.

## 6. Conclusion

There has been an increased focus on IS as a result of the COVID-19 pandemic and the significant impact on the global community. As the world moves beyond the pandemic, IS and the problems within the domain remain significant, with the growing threat of emerging infectious diseases, the global burden of sepsis, and the continual rise of AMR. The recent growth in the development of ML applications in the different areas of IS including the hospital, clinical laboratory, and public health setting has shown promising results. These applications have targeted a wide range of problems including the treatment and management of patients, the improvement of laboratory workflows, and predicting infection trends. However, there is a significant gap between the number of ML applications in research, and the effective implementation of ML systems. This paper has provided readers with an introduction to infection science, outlined the principles of trustworthy ML systems, presented an overview of the ML applications in IS that implement some of the principles of trustworthy AI, and identified the need for a change in focus towards implementing the practices of trustworthy ML systems to promote adoption of these systems in practice. Further work is required to ensure a more systematic view of trustworthiness is integrated into ML approaches in IS. In the future, we encourage researchers, engineers, and ML practitioners to address all areas of the ML system lifecycle, moving beyond model training and testing, and take a focused, end-to-end view of implementing trustworthy ML system principles in the IS domain to promote adherence to the regulatory requirements, stakeholder acceptance, and adoption of ML systems in practice within IS.

## References

[1] Muehlematter UJ Daniore P Vokinger KN. Approval of artificial intelligence, machine learning-based medical devices in the USA, Europe (2015–20): a comparative analysis. Lancet Digit Health. (2021) 3:e195–e203. 10.1016/S2589-7500(20)30292-2 33478929

[2] Inglis TJ. The foundations of aetiology: a common language for infection science. J Med Microbiol. (2023) 72:001637. 10.1099/jmm.0.001637 36917495

[3] Tsai JM Tolan NV Petrides AK Kanjilal S Brigl M Lindeman NI. How SARS-CoV-2 transformed the clinical laboratory: challenges, lessons learned. J Appl Lab Med. (2021) 6:1338–54. 10.1093/jalm/jfab034 33822967 PMC8083381

[4] Piret J Boivin G. Pandemics throughout history. Front Microbiol. (2021) 11:631736. 10.3389/fmicb.2020.631736 33584597 PMC7874133

[5] Laxminarayan R Duse A Wattal C Zaidi AKM Wertheim HFL Sumpradit N. Antibiotic resistance—the need for global solutions. Lancet Infect Dis. (2013) 13:1057–98. 10.1016/S1473-3099(13)70318-9 24252483

[6] Rudd KE Johnson SC Agesa KM Shackelford KA Tsoi D Kievlan DR. Global, regional,, national sepsis incidence, mortality, 1990–2017: analysis for the global burden of disease study. Lancet. (2020) 395:200–11. 10.1016/S0140-6736(19)32989-7 31954465 PMC6970225

[7] Huyen C. Designing machine learning systems: an iterative process for production-ready applications. O’Reilly Media, Incorporated (2022).

[8] He S Leanse LG Feng Y. Artificial intelligence, machine learning assisted drug delivery for effective treatment of infectious diseases. Adv Drug Deliv Rev. (2021) 178:113922. 10.1016/j.addr.2021.113922 34461198

[9] Tran NK Albahra S May L Waldman S Crabtree S Bainbridge S. Evolving applications of artificial intelligence, machine learning in infectious diseases testing. Clin Chem. (2021) 68:125–33. 10.1093/clinchem/hvab239 34969102 PMC9383167

[10] Peiffer-Smadja N Rawson T Ahmad R Buchard A Georgiou P Lescure FX. Machine learning for clinical decision support in infectious diseases: a narrative review of current applications. Clin Microbiol Infect. (2020) 26:584–95. 10.1016/j.cmi.2019.09.009 31539636

[11] Luz C Vollmer M Decruyenaere J Nijsten M Glasner C Sinha B. Machine learning in infection management using routine electronic health records: tools, techniques, and reporting of future technologies. Clin Microbiol Infect. (2020) 26:1291–9. 10.1016/j.cmi.2020.02.003 32061798

[12] Kamalov F Cherukuri A Sulieman H Thabtah F Hossain A. Machine learning applications for COVID-19: a state-of-the-art review [Preprint] (2021). Available at: 10.48550/arXiv.2101.07824

[13] Kushwaha S Bahl S Bagha AK Parmar KS Javaid M Haleem A. Significant applications of machine learning for COVID-19 pandemic. J Ind Integr Manag. (2020) 5:453–79. 10.1142/S2424862220500268

[14] Lalmuanawma S Hussain J Chhakchhuak L. Applications of machine learning and artificial intelligence for COVID-19 (SARS-CoV-2) pandemic: a review. Chaos Solitons Fractals. (2020) 139:110059. 10.1016/j.chaos.2020.110059 32834612 PMC7315944

[15] Syeda HB Syed M Sexton KW Syed S Begum S Syed F. Role of machine learning techniques to tackle the COVID-19 crisis: systematic review. JMIR Med Inform. (2021) 9:e23811. 10.2196/23811 33326405 PMC7806275

[16] Moor M Rieck B Horn M Jutzeler CR Borgwardt K. Early prediction of sepsis in the icu using machine learning: a systematic review. Front Med (Lausanne). (2021) 8:607952. 10.3389/fmed.2021.607952 34124082 PMC8193357

[17] Abdeldayem OM Dabbish AM Habashy MM Mostafa MK Elhefnawy M Amin L. Viral outbreaks detection and surveillance using wastewater-based epidemiology, viral air sampling, and machine learning techniques: A comprehensive review and outlook. Sci Total Environ. (2022) 803:149834. 10.1016/j.scitotenv.2021.149834 34525746 PMC8379898

[18] Bruyne SD Speeckaert MM Biesen WV Delanghe JR. Recent evolutions of machine learning applications in clinical laboratory medicine. Crit Rev Clin Lab Sci. (2021) 58:131–52. 10.1080/10408363.2020.1828811 33045173

[19] Peiffer-Smadja N Dellière S Rodriguez C Birgand G Lescure FX Fourati S. Machine learning in the clinical microbiology laboratory: has the time come for routine practice? Clin Microbiol Infect. (2020) 26:1300–9. 10.1016/j.cmi.2020.02.006 32061795

[20] Rabbani N Kim GY Suarez CJ Chen JH. Applications of machine learning in routine laboratory medicine: current state and future directions. Clin Biochem. (2022) 103:1–7. 10.1016/j.clinbiochem.2022.02.011 35227670 PMC9007900

[21] Agrebi S Larbi A. Use of artificial intelligence in infectious diseases. *Artificial intelligence in precision health*. Elsevier (2020). p. 415–438. 10.1016/b978-0-12-817133-2.00018-5

[22] Singer M Deutschman CS Seymour CW Shankar-Hari M Annane D Bauer M. The third international consensus definitions for sepsis and septic shock (sepsis-3). JAMA. (2016) 315:801–10. 10.1001/jama.2016.0287 26903338 PMC4968574

[23] Murray CJ Ikuta KS Sharara F Swetschinski L Robles Aguilar G Gray A. Global burden of bacterial antimicrobial resistance in 2019a systematic analysis. Lancet. (2022) 399:629–55. 10.1016/S0140-6736(21)02724-0 35065702 PMC8841637

[24] Talic S Shah S Wild H Gasevic D Maharaj A Ademi Z. Effectiveness of public health measures in reducing the incidence of COVID-19, SARS-CoV-2 transmission, COVID-19 mortality: systematic review, meta-analysis. BMJ. (2021) 375. 10.1136/bmj-2021-068302 34789505 PMC9423125

[25] Kelly CJ Karthikesalingam A Suleyman M Corrado G King D. Key challenges for delivering clinical impact with artificial intelligence. BMC Med. (2019) 17:1–9. 10.1186/s12916-019-1426-2 31665002 PMC6821018

[26] High-Level Expert Group on AI. *Ethics guidelines for trustworthy AI*. Brussels: European Commission(2019). Report.

[27] Zicari RV Brusseau J Blomberg SN Christensen HC Coffee M Ganapini MB. On assessing trustworthy ai in healthcare. Machine learning as a supportive tool to recognize cardiac arrest in emergency calls. Front Hum Dyn. (2021) 3:30. 10.3389/fhumd.2021.673104

[28] Pruksachatkun Y Mcateer M Majumdar S. Practicing trustworthy machine learning. O’Reilly Media (2023).

[29] Varshney KR. Trustworthy machine learning. Chappaqua, NY: Independently Published (2022).

[30] Li B Qi P Liu B Di S Liu J Pei J. Trustworthy AI: from principles to practices. ACM Comput Surv. (2023) 55:1–46. 10.1145/3555803

[31] Zicari RV Brodersen J Brusseau J Düdder B Eichhorn T Ivanov T. Z-inspection®: a process to assess trustworthy ai. IEEE Trans Technol Soc. (2021) 2:83–97. 10.1109/TTS.2021.3066209

[32] Zicari RV Ahmed S Amann J Braun SA Brodersen J Bruneault F. Co-design of a trustworthy ai system in healthcare: deep learning based skin lesion classifier. Front Hum Dyn. (2021) 3:688152. 10.3389/fhumd.2021.688152

[33] Allahabadi H Amann J Balot I Beretta A Binkley C Bozenhard J. Assessing trustworthy AI in times of COVID-19. Deep learning for predicting a multi-regional score conveying the degree of lung compromise in COVID-19 patients. IEEE Trans Technol Soc. (2022) 3. 10.1109/TTS.2022.3195114 PMC9762021 36573115

[34] Baker-Brunnbauer J. TAII framework for trustworthy AI systems. ROBONOMICS: J Autom Econ. (2021) 2:17. https://ssrn.com/abstract=3914105

[35] Ala-Pietilä P Bonnet Y Bergmann U Bielikova M Bonefeld-Dahl C Bauer W. The assessment list for trustworthy artificial intelligence (ALTAI). European Commission (2020).

[36] Scott I Carter S Coiera E. Clinician checklist for assessing suitability of machine learning applications in healthcare. BMJ Health Care Inform. (2021) 28. 10.1136/bmjhci-2020-100251 PMC7871244 33547086

[37] Hu C Li L Huang W Wu T Xu Q Liu J. Interpretable machine learning for early prediction of prognosis in sepsis. A discovery and validation study. Infect Dis Ther. (2022) 11:1–16. 10.1007/s40121-022-00628-6 35399146 PMC9124279

[38] Lundberg SM Lee SI. A unified approach to interpreting model predictions. In: Guyon I, Luxburg UV, Bengio S, Wallach H, Fergus R, Vishwanathan S, et al., editors. *Advances in Neural Information Processing Systems 30*. Curran Associates, Inc. (2017). p. 4765–4774.

[39] Nemati S Holder A Razmi F Stanley MD Clifford GD Buchman TG. An interpretable machine learning model for accurate prediction of sepsis in the ICU. Crit Care Med. (2018) 46:547. 10.1097/CCM.0000000000002936 29286945 PMC5851825

[40] Jiang Z Bo L Xu Z Song Y Wang J Wen P. An explainable machine learning algorithm for risk factor analysis of in-hospital mortality in sepsis survivors with ICU readmission. Comput Methods Programs Biomed. (2021) 204:106040. 10.1016/j.cmpb.2021.106040 33780889

[41] Casiraghi E Malchiodi D Trucco G Frasca M Cappelletti L Fontana T. Explainable machine learning for early assessment of COVID-19 risk prediction in emergency departments. IEEE Access. (2020) 8:196299–325. 10.1109/ACCESS.2020.3034032 34812365 PMC8545262

[42] Tabaie A Orenstein EW Nemati S Basu RK Kandaswamy S Clifford GD. Predicting presumed serious infection among hospitalized children on central venous lines with machine learning. Comput Biol Med. (2021) 132:104289. 10.1016/j.compbiomed.2021.104289 33667812 PMC9207586

[43] Das AK Mishra S Gopalan SS. Predicting COVID-19 community mortality risk using machine learning and development of an online prognostic tool. PeerJ. (2020) 8:e10083. 10.7717/peerj.10083 33062451 PMC7528809

[44] Duckworth C Chmiel FP Burns DK Zlatev ZD White NM Daniels TW. Using explainable machine learning to characterise data drift and detect emergent health risks for emergency department admissions during COVID-19. Sci Rep. (2021) 11:1–10. 10.1038/s41598-021-02481-y 34837021 PMC8626460

[45] González-Gonzalo C Thee EF Klaver CC Lee AY Schlingemann RO Tufail A. Trustworthy AI: closing the gap between development and integration of AI systems in ophthalmic practice. Prog Retin Eye Res (2021) 90:101034. 10.1016/j.preteyeres.2021.101034 34902546 PMC11696120

[46] Beltrão G Paramonova I Sousa S. User interface design for AI-based clinical decision-support system: preliminary study. *2022 17th Iberian Conference on Information Systems and Technologies (CISTI)* (2022). p. 1–4. Available from: 10.23919/CISTI54924.2022.9820378

[47] Liao Q Sundar SS. Designing for responsible trust in ai systems: a communication perspective. *Proceedings of the 2022 ACM Conference on Fairness, Accountability, and Transparency*. New York, NY, USA: Association for Computing Machinery (2022). FAccT ’22. p. 1257–1268. 10.1145/3531146.3533182

[48] Bach TA Khan A Hallock H Beltrão G Sousa S. A systematic literature review of user trust in AI-enabled systems: an HCI perspective. Int J Hum-Comput Interact. (2022) 1–16. 10.1080/10447318.2022.2138826

